# *Irf5* deficiency in myeloid cells prevents necrotizing enterocolitis by inhibiting M1 macrophage polarization

**DOI:** 10.1038/s41385-019-0169-x

**Published:** 2019-05-13

**Authors:** Jia Wei, Daxing Tang, Chengjie Lu, Jin Yang, Yulei Lu, Yidong Wang, Liangliang Jia, Jianfang Wang, Wei Ru, Yi Lu, Zhejun Cai, Qiang Shu

**Affiliations:** 1 Children’s Hospital, Zhejiang University School of Medicine, Hangzhou, China; 2 Center for Translational Medicine, the Affiliated Hospital of Hangzhou Normal University, Hangzhou, China; 3 Department of Cardiology, the Second Affiliated Hospital, Zhejiang University School of Medicine, Hangzhou, China; 4 The Key Laboratory of Cardiovascular Remodeling and Function Research, Chinese Ministry of Education and Chinese Ministry of Health, and The State and Shandong Province Joint Key Laboratory of Translational Cardiovascular Medicine, Qilu Hospital of Shandong University, Jinan, China

## Abstract

Necrotizing enterocolitis (NEC) is a life-threatening inflammatory disease in newborns, but the mechanisms remain unclear. Interferon regulatory factor 5 (IRF5) is a master regulator of macrophage function and is essential for proinflammatory M1 macrophage polarization. Our previous data indicated that M1 macrophages promote NEC injury. Here, we investigated whether IRF5 is involved in the pathogenesis of NEC. First, we found that IRF5 was upregulated in infiltrated macrophages in human neonates with NEC compared to controls. We further confirmed IRF5 upregulation in macrophages in experimental murine NEC and that the infiltrated macrophages were predominantly polarized into the M1 but not the M2 phenotype. Myeloid-specific deficiency of *Irf5*, which was associated with reduced M1 macrophage polarization and systematic inflammation, dramatically prevented experimental NEC. Moreover, we found that the ablation of *Irf5* in myeloid cells markedly suppressed intestinal epithelial cell apoptosis and further prevented intestinal barrier dysfunction in experimental NEC. Bioinformatic and chromatin immunoprecipitation analysis further showed that IRF5 binds to the promoters of the M1 macrophage-associated genes *Ccl4*, *Ccl5*, *Tnf*, and *Il12b*. Overall, our study provides evidence that IRF5 participates in the pathogenesis of NEC, while the deletion of *Irf5* in myeloid cells prevents NEC via inhibiting M1 macrophage polarization.

## Introduction

Necrotizing enterocolitis (NEC) is a severe inflammatory disease that affects the gastrointestinal tract of premature infants and is the most common cause of gastrointestinal mortality in newborns.^1^ The incidence of NEC has been determined to be as high as 13% among infants born at ≤33 gestational weeks or with a birth weight ≤ 2500 g.^2^ Moreover, the average mortality from NEC is 20–30% and as high as 50% in infants who require surgical intervention.^3^ Approximately half of NEC survivors suffer from early postoperative complications, such as wound infection, breakdown or dehiscence, and long-term complications including intestinal stricture and short-gut syndrome.^4^ Despite several decades of studies on the pathogenesis of NEC, the detailed mechanisms remain incompletely understood.

Several factors including prematurity, microbial immaturity, intestinal barriers and the response to inflammation by the premature intestine are considered to be involved in the pathogenesis of NEC.^5^ One of the critical pathological features of NEC is the accumulation of rich inflammatory cells that infiltrate the intestinal mucosa.^6,7^ Studies have indicated that macrophages play an essential role in the development of NEC.^7^ The premature innate immune system is associated with a hyperinflammatory intestinal macrophage phenotype that causes increased NEC injury.^8^ Furthermore, macrophages promote nuclear factor (NF)-κB-mediated inflammatory signaling in NEC through toll-like receptors (TLRs) activation.^6,9^

Interferon regulatory factor 5 (IRF5) which is mainly expressed in immune cells, is involved in TLRs-mediated signal transduction and immune cell differentiation.^10^ It has been revealed that IRF5 is crucial in the development of various autoimmune diseases, such as rheumatoid arthritis^11^ and systemic lupus erythematosus.^12^ Recent studies have further suggested that IRF5 plays a key role in polarizing macrophages towards a pro-inflammatory M1 phenotype,^13,14^ and that IRF5 induction in macrophages promotes the pathogenesis of various inflammatory diseases including sepsis,^13,15^ insulin resistance during obesity,^16^ myocardial infarction,^17^ and atherosclerosis.^18^ However, the role of IRF5 in NEC has never been explored.

It has been recently reported that IRF5 acts as a key regulator of M1 macrophage responses in newborns,^19^ and our previous report confirmed that M1 macrophages promote NEC.^20^ Regarding the crucial role of IRF5 in M1 macrophage polarization, we hypothesized that *Irf5* deficiency in macrophages may prevent NEC. To test this hypothesis, we generated myeloid-specific *Irf5*-deficient mice and investigated the effect on NEC.

## Materials and methods

### Human specimen of NEC collection

All the studies followed the guidelines for the ethical treatment of human specimen and were approved by the Children’s Hospital, Zhejiang University School of Medicine. According to the Declaration of Helsinki, written informed consents were obtained from the parents of all the neonates. Neonates with complicated NEC (Bell stage III) or congenital intestinal atresia were selected for surgery by neonatologists and pediatric surgeons, per standard of care. Bell’s classification, which categorizes cases of NEC into stage I, II, or III, is widely used to assess the degree of NEC severity in the clinic.^2^ Surgical resections of necrotizing small intestines from NEC patients, as well as pieces of normal small intestines from the edge of the atresia segment in the congenital intestinal atresia, which served as controls, were harvested, rinsed in PBS, fixed in 4% paraformaldehyde in PBS, and embedded in paraffin for histopathological analyses. Detailed characteristics of the subjects are listed in Supplemental Table 1.

### Mice

C57BL/6J mice were purchased from Shanghai Slac Laboratory Animal Co. Ltd. (Shanghai, China). *Irf5*^fl/fl^ mice were purchased from Jackson Laboratory (Bar Harbor, ME), and *Lyz2-Cre* mice were purchased from Nanjing BioMedical Research Institute of Nanjing University, China. Myeloid cell-specific *Irf5* knockout mice (*Irf5*^ΔMΦ^) were created by crossing the *Irf5*^fl/fl^ mice with the *Lyz2-Cre* transgenic mice. Littermates not carrying the *Lyz2-Cre* transgene (*Irf5*^fl/fl^) served as controls. All the mice were on a C57BL/6J background.

### Murine model of NEC

The following experimental protocols followed the guidelines for the ethical treatment of experimental animals as approved by the Institutional Animal Care and Use Committee of the Zhejiang University School of Medicine. Pups were randomized into the following groups: (1) WT breast-fed (Vehicle) (*n* = 5) and (2) WT NEC (*n* = 13). Similarly, the pups of the *Irf5*^fl/fl^ and *Irf5*^ΔMΦ^ mice were randomized into the following groups: (1) *Irf5*^fl/fl^ breast-fed (Vehicle) (*n* = 5); (2) *Irf5*^fl/fl^ NEC (*n* = 13); (3) *Irf5*^ΔMΦ^ breast-fed (Vehicle) (*n* = 5); and (4) *Irf5*^ΔMΦ^ NEC (*n* = 12).

NEC was induced using a modified version of the model we had previously established.^21^ Briefly, the pups were collected after vaginal delivery and prior to breast-feeding. They were recovered, dried and placed in an incubator at 35 °C. Pups were fed Similac 60/40 formula (Ross Pediatrics, Columbus, OH) fortified with Esbilac powder (Pet-Ag, New Hampshire, IL), which provided 836.8 kJ/kg per day as previously described.^21^ The pups were exposed to hypoxia (95% nitrogen for 1 min) followed by hypothermia (4 °C for 10 min) every 12 h. The pups were sacrificed upon the development of the clinical signs of NEC, which included abdominal distention, bloody stools or respiratory distress. Any surviving pups were sacrificed 96 h after birth. The intestines were harvested immediately upon sacrifice. All pups were included in analyses.

### Histologic injury grading

The small intestines from the pups were fixed in 4% paraformaldehyde in PBS and embedded in paraffin, and hematoxylin and eosin–stained sections were obtained. Intestinal injury was measured using a standard histological scoring system in which injury was graded as follows:^21^ grade 0, normal intestine; grade 1, epithelial cell lifting or separation; grade 2, necrosis to the mid villus level; grade 3, necrosis of the entire villus; and grade 4, transmural necrosis. Any injury of grade 2 or above was considered consistent with NEC. Five intestinal segments from each mouse were analyzed. Twenty-four fields from each section were viewed and graded blindly by two independent investigators.

### Immunohistochemistry and immunofluorescence

The human intestinal tissue samples were analyzed by immunohistochemical staining using an anti-IRF5 antibody (1:200, ab181553) from Abcam (United Kingdom). Immunofluorescence was performed by double staining with an anti-IRF5 antibody (1:200, ab181553) from Abcam and an anti-CD68 antibody (1:200, MCA1957GA) from BioRad (Hercules, CA). The murine intestinal tissues were stained with an anti-IRF5 antibody (1:200, ab181553) from Abcam, an anti-CD68 antibody (1:200, MCA1957GA) from BioRad, and an anti-iNOS antibody (1:200, ab15323) from Abcam. Five serial histologic sections per pup were examined.

### Western blotting

Intestinal tissues were harvested and lysed in RIPA lysis buffer containing protease inhibitors. An equal amount of protein from all the samples was separated by SDS-PAGE and then transferred to PVDF membranes. The following antibodies were applied: anti-IRF5 (1:1000, ab181553) from Abcam and anti-cleaved caspase-3 (1:1000, 9661S) and anti-GAPDH (1:3000, 3668S) from Cell Signaling Technology (Danvers, MA).

### Quantitative PCR

Total RNA was extracted from whole intestinal tissues using Trizol reagent (Invitrogen) and subsequently reversely transcribed into cDNA using a PrimeScript RT reagent kit (RR037A, Takara, Japan) according to the manufacturer’s instructions. The cDNA was subjected to real-time PCR using a SYBR Premix Ex Taq II (Tli RNaseH Plus) kit (RR820A, Takara) and an Applied Biosystems 7500 Fast Real-Time PCR System (ABI, Torrance, CA). GAPDH was used as an internal control. The primer information can be found in Supplemental Table 2.

### TUNEL staining

Cryosections of the intestinal tissues were fixed with 4% (w/v) paraformaldehyde in PBS for 15 min, and apoptosis was quantified by using the TUNEL assay with the In Situ Cell Death Detection Kit, TMR red (12156792910, Roche, Indianapolis, IN) according to the manufacturer’s instructions. Anti-EpCAM (1:200, sc-53532) from Santa Cruz Biotechnology (Santa Cruz, CA) was used to label the intestinal epithelial cells (IECs). Four random high-power fields per section and 3 sections per pup were analyzed.

### ELISA

Serum IL-1β and TNF-α levels were assayed by ELISA kits (USCN Business Co., Ltd, China) according to the manufacturer’s instructions.

### Nitrite assay

Serum nitrite levels were determined by Griess reagent (G2930, Promega, Madison, WI) according to the manufacturer’s protocol. The absorbance of each sample was measured at 520 nm using a spectrophotometer.

### Intestinal permeability

Fluorescein isothiocyanate (FITC)-labeled dextran molecules (FD70, molecular weight 73,000; Sigma-Aldrich, St. Louis, MI, 750 mg/ml) was administered enterally 4 h prior to euthanasia. The serum concentration of FITC-dextran was measured by a fluorescent plate reader with a 492/515 nm filter set.

### Bioinformatic analysis of the ChIP-Seq dataset

The chromatin immunoprecipitation-sequencing (ChIP-Seq) dataset E-MTAB-2661 comparing WT and *Irf5*^−/−^ bone marrow-derived macrophages (BMDMs) following stimulation with LPS for 2 h were analyzed.^22^ The reads were aligned to the mouse genome (mm9) using Bowtie (version 1.2.1) software. After format conversion and sorting, duplicate reads were removed by the rmdup tool from the SAMtools package. To identify the binding sites of IRF5, the mapped sequence reads were processed with MACS version 1.4.2 against their matching control samples. Only peaks with *P* values < 10^−5^ were kept for further analyses. The annotatePeaks.pl Perl script of the HOMER package was used to associate the ChIP peaks with nearby genes, and the location and the shape of the called peaks was visualized using Integrative Genomics Viewer (IGV).^23^

### Cell culture and treatment

BMDMs were prepared as described previously.^24^ Bone marrow of the femur and tibia of C57BL/6J mice was flushed with PBS. The cells were cultured in RPMI-1640 medium supplemented with 10% FBS and mouse M-CSF recombinant protein (50 ng/ml, eBioscience, Waltham, MA) for 5–7 days. The cells were left unstimulated or were stimulated with LPS (100 ng/ml, Escherichia coli 0111:B4, Sigma-Aldrich).

### ChIP-quantitative PCR

ChIP assays were performed with a kit (Millipore, Billerica, MA) according to the manufacturer’s instructions and as described previously.^25^ A total of 1 × 10^7^ BMDMs were lysed and sonicated to break up the DNA into 100–500-bp fragments. The target proteins were immunoprecipitated overnight at 4 °C with protein A-conjugated Sepharose beads in the presence of an antibody against IRF5 from Abcam (ab21689). All the DNA samples were purified using a Universal DNA Purification Kit (TIANGEN Biotech, China) before PCR analysis. PCR was conducted using PrimeSTAR HS DNA polymerase (premix) to amplify the fragment surrounding the binding sites of interest (TaKaRa, Japan). The primers used to amplify the binding regions of the indicated gene promoters are listed in Supplemental Table 3.

### Statistical analysis

The data are presented as the mean ± standard deviation (SD) or the median (25th–75th percentile). One-way analysis of variance (ANOVA), Student’s *t* test, or the Mann–Whitney test was used to compare the means of multiple groups, and the *χ*2 test was used to compare categorical variables. *P* values of < 0.05 were considered statistically significant. All of the statistical calculations were carried out using SPSS version 17.0 (SPSS Inc, Chicago, IL, USA).

## Results

### IRF5 is upregulated in macrophages in human neonates with NEC

**Fig. 1:**
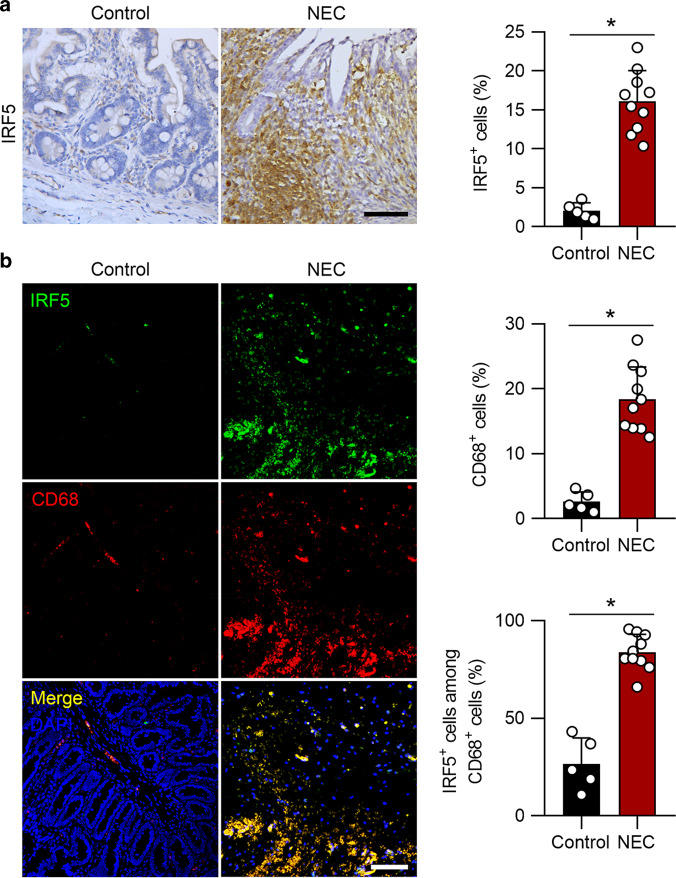
IRF5 is induced in infiltrated macrophages in human NEC neonates. **a** Representative immunohistochemical staining for IRF5 in the intestines of control and NEC neonates. NEC neonates exhibited a significant elevation in IRF5 expression. **b** Representative immunofluorescence staining for IRF5 (green) and CD68 (red) in control and NEC intestines. NEC led a significantly increased number of CD68-positive cells in the intestines, and IRF5 was mostly localized in CD68-positive cells. (*n* = 5 in the control group; *n* = 10 in the NEC group. Scale bar: 50 μm in (**a**) and 100 μm in (**b**). *P < 0.05)

We first explored whether IRF5 is involved in NEC. Intestinal samples were collected from neonates with Bell stage III NEC or from patients with congenital intestinal atresia, who served as controls. Immunochemical staining revealed significant upregulation of IRF5 in human clinical samples of NEC patients compared to the controls (Fig. 1a). Moreover, immunofluorescence analysis further showed a marked increase in the number of CD68-positive cells coexpressing IRF5, indicating that IRF5 was upregulated in infiltrated macrophages in neonates with NEC (Fig. 1b).

### IRF5 upregulation is accompanied by increased M1 macrophage polarization in murine experimental NEC

**Fig. 2:**
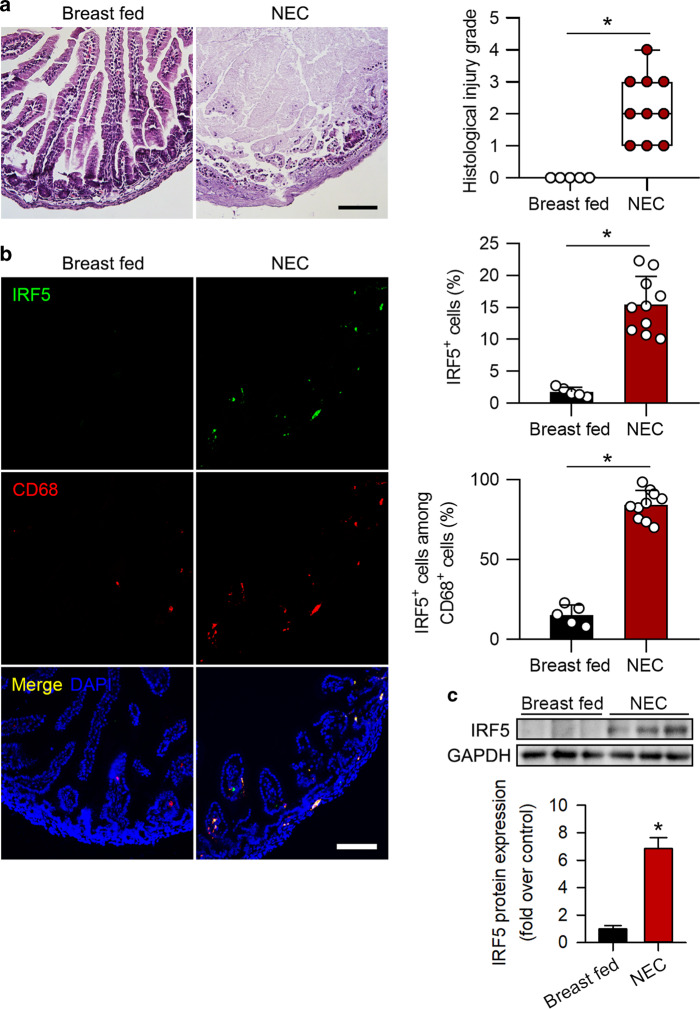
IRF5 is induced in infiltrated macrophages in murine experimental NEC. **a** Representative HE staining of samples from breast-fed pups and pups with NEC. Histological injury grade was significantly increased in the NEC group. **b** Representative immunofluorescence staining for IRF5 (green) and CD68 (red) in the intestines of mouse pups. The pups with NEC exhibited significantly increased IRF5 expression compared to that in the breast-fed pups, and IRF5 was mostly localized in infiltrated CD68-positive cells in the intestines. **c** Western blotting analysis showed that IRF5 expression in the intestine was significantly induced in the NEC pups compared to the breast-fed pups. (*n* = 5 in breast fed; *n* = 10 in NEC. Scale bar: 100 μm. *P < 0.05)

We measured IRF5 expression in a murine model of experimental NEC. NEC was successfully induced, as shown by increased NEC scores (Fig. 2a). As predicted, IRF5 expression was markedly increased in the pups with experimental NEC compared to the control pups (Fig. 2b, c). The immunofluorescence staining results further confirmed that the increase in IRF5 in NEC was localized to infiltrated macrophages (Fig. 2b).

**Fig. 3:**
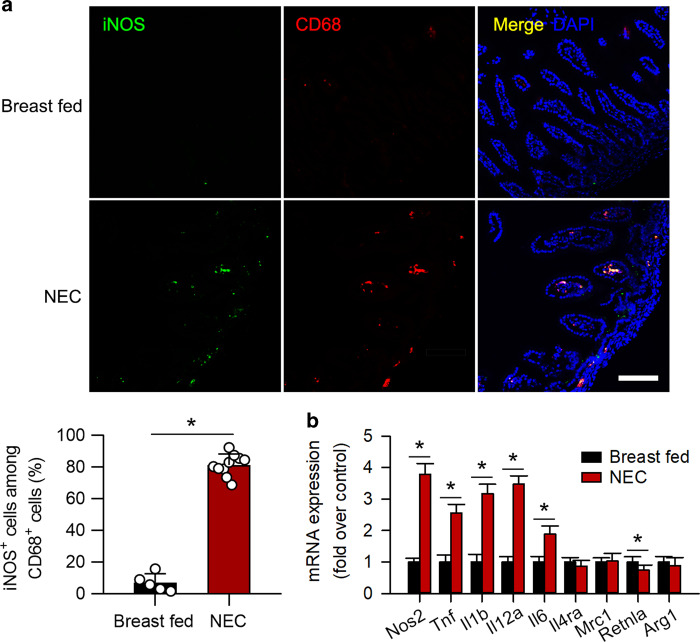
Infiltrated macrophages are polarized into the M1 phenotype in experimental NEC. **a** Representative immunofluorescence staining for iNOS (green) and CD68 (red) in the intestine. The pups with NEC exhibited a significant increase in the number of iNOS^+^CD68^+^ cells in the intestines compared to that in breast-fed pups. **b** Quantitative PCR analysis of the mRNA expression of M1 and M2 macrophage-associated genes in the intestines. The mRNA expression of M1 macrophage-associated genes was significantly increased in NEC, while the mRNA expression of M2 macrophage-associated genes, except that of *Retnla*, was unchanged. (*n* = 5 in the breast-fed group; *n* = 10 in the NEC group. *P < 0.05)

It has been documented that IRF5 mediates macrophage M1 polarization,^26^ and our previous report has suggested that M1 macrophages promote NEC.^20^ Immunofluorescence staining showed that the expression of a M1 macrophage marker, iNOS, was significantly induced and that iNOS was mostly colocalized with CD68 (Fig. 3a). Quantitative PCR analysis further revealed that the mRNA expression of M1 macrophage-associated genes including *Tnf*, *Il1b*, *Il6*, *Il12a*, and *Nos2* were upregulated, while the expression of M2 macrophage-associated genes, except that of *Retnla*, was not significantly changed (Fig. 3b). These data indicate that M1 but not M2 macrophage polarization is triggered in experimental NEC.

### Myeloid-specific deficiency of *Irf5* prevents intestinal injury in experimental NEC

**Fig. 4:**
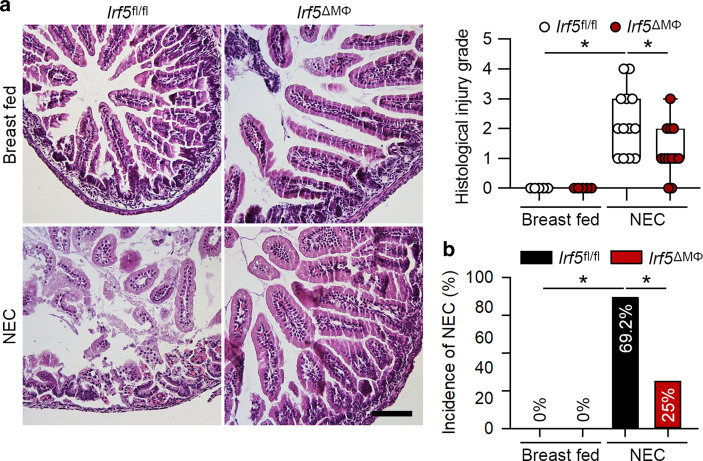
Myeloid-specific deficiency of *Irf5* attenuates experimental NEC. **a** Representative HE staining of the intestines of the indicated groups. The *Irf5*^fl/fl^ pups with NEC exhibited a significantly increased histological injury grade compared to that of the breast-fed pups, while pups with myeloid-specific *Irf5* deletion exhibited a markedly reduced histological injury grade compared to that in the *Irf5*^fl/fl^ pups with NEC. **b** The pups with myeloid-specific ablation of *Irf5* exhibited a significantly reduced incidence of NEC compared to that in the *Irf5*^fl/fl^ pups with NEC (*n* = 5 in the breast-fed *Irf5*^fl/fl^ group; *n* = 5 in the breast-fed *Irf5*^ΔMΦ^ group; *n* = 13 in the NEC *Irf5*^fl/fl^ group; *n* = 12 in the NEC *Irf5*^ΔMΦ^ group. Scale bar: 100 μm. *P < 0.05)

Our data showed that IRF5 expression was dramatically upregulated in macrophages in NEC, we therefore determined the effect of *Irf5* deficiency in myeloid cells in experimental NEC. The pups of the *Irf5*^fl/fl^ mice exposed to experimental NEC exhibited a significantly increased histologic injury grade compared to that of the breast-fed *Irf5*^fl/fl^ pups (Fig. 4). However, the pups of the *Irf5*^ΔMΦ^ mice, which lack *Irf5* in myeloid cells, that were exposed to NEC exhibited a significant reduction in histologic injury grade and the incidence of NEC compared to those of the *Irf5*^fl/fl^ pups with NEC (Fig. 4).

### Myeloid-specific deletion of *Irf5* prevents M1 macrophage polarization in experimental NEC

**Fig. 5:**
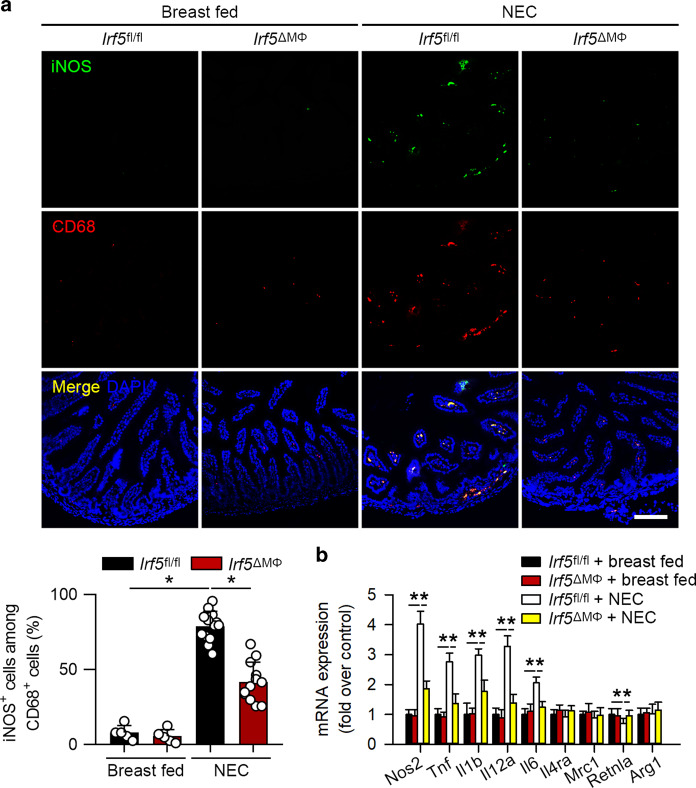
*Irf5* ablation in myeloid cells suppresses M1 macrophage polarization in experimental NEC. **a** Representative immunofluorescence staining for iNOS (green) and CD68 (red) in the intestines of the indicated groups. The pups with NEC exhibited a significant increase in the number of iNOS^+^CD68^+^ cells in the intestines compared to that in the breast-fed pups, while *Irf5* deletion in myeloid cells markedly blocked this effect. **b** Quantitative PCR analysis of the mRNA expression of M1 and M2 macrophage-associated genes in the intestines of the indicated groups. The myeloid-specific deficiency of *Irf5* significantly inhibited the NEC-induced mRNA expression of M1 macrophage-associated genes in the *Irf5*^fl/fl^ pups. The mRNA expression of the M2 macrophage-associated genes, except that of *Retnla*, was unaltered among the groups. (*n* = 5 in the breast-fed *Irf5*^fl/fl^ group; *n* = 5 in the breast-fed *Irf5*^ΔMΦ^ group; *n* = 13 in NEC *Irf5*^fl/fl^ group; *n* = 12 in the NEC *Irf5*^ΔMΦ^ group. Scale bar: 100 μm. *P < 0.05)

We next evaluated whether *Irf5* deletion in myeloid cells could suppress M1 macrophage polarization in NEC. NEC significantly increased iNOS staining in CD68-positive cells in the *Irf5*^fl/fl^ mice (Fig. 5a). However, the increased iNOS staining in CD68-positive cells was significantly blocked in the *Irf5*^ΔMΦ^ mice with NEC (Fig. 5a). In addition, *Irf5* ablation in myeloid cells markedly attenuated the NEC-induced mRNA expression of M1 macrophage-associated genes, as assessed by quantitative PCR (Fig. 5b). These data suggest that *Irf5* deficiency in myeloid cells inhibits M1 macrophage polarization in NEC.

### Decreased inflammation in myeloid *Irf5*-deficient pups exposed to experimental NEC

Abundant evidence suggest that M1 macrophages produce inflammatory cytokines that drive pro-inflammatory cascades.^27,28^ Serum levels of IL-1β and TNF-α, as determined by ELISA, were significantly upregulated in *Irf5*^fl/fl^ pups with NEC (Supplemental Fig. 1). However, the myeloid-specific deficiency of *Irf5* significantly attenuated the upregulation of cytokines induced by experimental NEC (Supplemental Fig. 1). We also tested serum nitrite levels, which indicate iNOS activity. As expected, NEC challenge led to a robust induction of serum nitrite levels, while the myeloid-specific deficiency of *Irf5* markedly suppressed this effect (Supplemental Fig. 2).

### *Irf5* ablation in myeloid cells attenuates intestinal epithelial cell apoptosis and intestinal barrier dysfunction

**Fig. 6:**
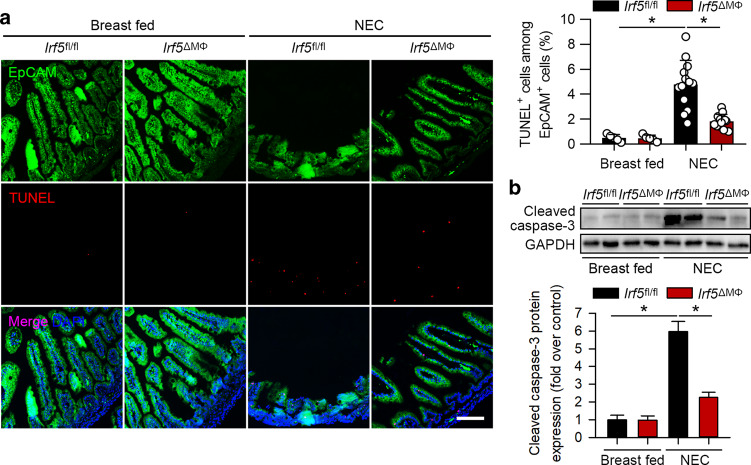
Myeloid-specific deletion of *Irf5* inhibits intestinal epithelial cell apoptosis in experimental NEC. **a** Representative TUNEL (red) staining of the intestines of the indicated groups. Intestinal epithelial cells were stained with EpCAM (green). NEC significantly increased TUNEL^+^EpCAM^+^ cells in the *Irf5*^fl/fl^ pups, while *Irf5* deletion in myeloid cells markedly blocked this effect. **b** Western blotting analysis of cleaved caspase-3 expression in the intestines of the indicated groups. The expression of cleaved caspase-3 was significantly increased in the *Irf5*^fl/fl^ pups with NEC compared to the breast-fed *Irf5*^fl/fl^ pups, while *Irf5* deletion in myeloid cells significantly suppressed this effect. (*n* = 5 in the breast-fed *Irf5*^fl/fl^ group; *n* = 5 in the breast-fed *Irf5*^ΔMΦ^ group; *n* = 13 in the NEC *Irf5*^fl/fl^ group; *n* = 12 in the NEC *Irf5*^ΔMΦ^ group. Scale bar: 100 μm. *P < 0.05)

Apoptosis in IECs is responsible for intestinal barrier dysfunction and contributes to NEC development.^29^ We performed TUNEL analysis to assay apoptosis in IECs. As shown in Fig. 6a, the *Irf5*^fl/fl^ pups exposed to NEC exhibited a significant induction of TUNEL-positive staining in EpCAM-positive IECs. However, TUNEL staining in IECs was significantly reduced in the *Irf5*^ΔMΦ^ pups exposed to NEC compared to the *Irf5*^fl/fl^ pups with NEC (Fig. 6a). Moreover, NEC significantly induced cleaved caspase-3 expression, but cleaved caspase-3 expression was markedly suppressed by *Irf5* deficiency in myeloid cells (Fig. 6b).

We further tested intestinal barrier function in the mice. The pups of the *Irf5*^fl/fl^ mice exposed to NEC exhibited significantly enhanced intestinal permeability of FITC-dextran compared to that exhibited by the breast-fed *Irf5*^fl/fl^ pups (Supplemental Fig. 3). The intestinal permeability of FITC-dextran in the *Irf5*^ΔMΦ^ pups subjected to NEC was markedly attenuated compared to that in the *Irf5*^fl/fl^ pups with NEC (Supplemental Fig. 3).

### IRF5 regulates M1 macrophage-associated genes

**Fig. 7:**
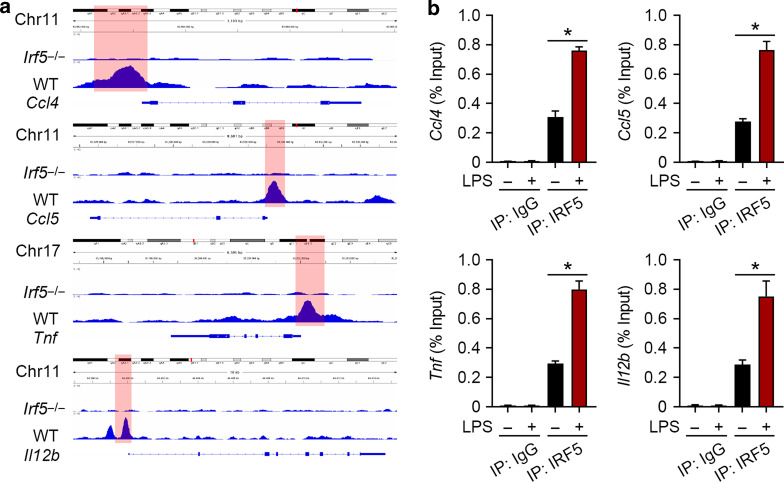
IRF5 binds with the promoters of M1 macrophage-associated genes. **a** Representative IGV tracks in the *Ccl4*, *Ccl5*, *Tnf*, and *Il12b* loci for IRF5 of LPS-stimulated (2 h) WT and *Irf5*^-/-^ BMDMs. **b** ChIP assays were performed to analyze the interaction between IRF5 and the *Ccl4*, *Ccl5*, *Tnf*, *Il12b* promoters. Quantitative PCR analysis showed that IRF5 binds with the promoter regions of *Ccl4*, *Ccl5*, *Tnf*, and *Il12b*. LPS significantly promoted these bindings (*n* = 3, *P < 0.05)

To investigate the regulatory role of IRF5 in M1 macrophage polarization and biological function, bioinformatic analysis of a ChIP-Seq dataset (E-MTAB-2661^22^) was applied, for which WT and *Irf5*^-/-^ BMDMs were treated with LPS for 2 h. The illustrative binding regions of the M1 macrophage-associated genes *Ccl4*, *Ccl5*, *Tnf*, and *Il12b* are shown Fig. 7a. Untreated BMDMs or those treated with LPS for 2 h were used for ChIP, which confirmed that LPS treatment induced IRF5 binding in the promoter regions of these M1 macrophage-associated genes (Fig. 7b).

## Discussion

Macrophages are powerful contributors to the development of NEC,^7,30^ and IRF5 is a master regulator of macrophage function.^31^ In the present study, we found that IRF5 is induced in NEC, and the deletion of *Irf5* in myeloid cells significantly prevents NEC in mice. *Irf5* ablation suppresses the M1 macrophage polarization induced by NEC. This leads to a significant reduction in inflammatory responses and barrier dysfunction in NEC.

Abundant reports have linked IRF5 to inflammatory diseases.^31,32^ IRF5 not only plays a vital role in regulating type I interferon expression induced by viruses and pathogens^33,34^ but has recently gained much attention as an immune regulator of inflammatory responses and autoimmune diseases as well.^35,36^ Upon the activation of TLRs by various stimuli, IRF5 participates in promoting downstream pro-inflammatory signaling.^10^ Considering the important role of TLRs in NEC,^37,38^ it is reasonable that IRF5 may also be induced in NEC. Indeed, our data show that IRF5 is upregulated in NEC and, more importantly, that it is mainly induced in infiltrated macrophages.

Macrophages are essential immune cells in the innate immune system. Investigations have identified the pathogenic role of macrophages in NEC in producing inflammatory cytokines and impairing intestinal barrier function.^7,8^ It has been well-documented that macrophages can undergo polarization into a classically activated proinflammatory M1 phenotype or an alternatively activated anti-inflammatory M2 phenotype.^39^ During NEC, disturbed barrier function leads to an imbalanced intestinal microbe environment with increased levels of LPS and proinflammatory cytokines,^40^ which are classical stimuli of M1 macrophage polarization.^41^ Indeed, we identified M1 macrophages as the predominant macrophages in experimental NEC.

IRF5 serves as a master regulator of macrophage polarization.^13^ It senses danger signals and induces inflammatory gene expression, giving rise to M1 macrophages.^10^ The silencing or a deficiency of *Irf5* hampers M1 macrophage polarization,^1^ and in our study, we also confirmed that the myeloid-specific ablation of *Irf5* reduces M1 macrophage polarization in the intestines of mice affected by NEC.

Our previous work has implicated the participation of M1 macrophages in NEC by promoting inflammation and intestinal epithelial cell apoptosis.^20^ This implies that, by inhibiting M1 macrophage polarization, it may be possible to suppress NEC in vivo. In fact, considerable studies have reported that modulating macrophage polarization, especially inhibiting M1 macrophage polarization, shows protective effects in inflammatory diseases such as ulcerative colitis.^42^ Regarding IRF5, various reports have shown that *Irf5* ablation, especially in myeloid cells, inhibits M1 macrophage polarization and suppresses the progression of inflammatory diseases such as sepsis^31^ and systemic lupus erythematosus.^35^ Moreover, a recent study reported that the silencing of *Irf5* by nanoparticle-delivered siRNA improved myocardial infarction by reducing M1 macrophage polarization in vivo.^17^ Our data show that the myeloid-specific deficiency of *Irf5* prevents M1 macrophage polarization, which further reduces overactivated inflammatory responses, intestinal epithelial cell apoptosis and barrier dysfunction in NEC.

Abundant evidence has suggested the presence of increased concentrations of LPS as well as TLRs/MyD88-associated proinflammatory cytokines including IL-1β, IL-6, and TNF-α in the serum of patients of NEC.^43,44^ In animal models, the results are extremely similar, that is, proinflammatory cytokines such as IL-1β, IL-6, and TNF-α are upregulated.^45^ Our cellular data show that IRF5 directly binds with the promoter regions of the M1 macrophage-associated genes *Ccl4*, *Ccl5*, *Tnf* and *Il12b*, which are induced by LPS. Moreover, *Irf5* ablation in myeloid cells suppresses serum proinflammatory cytokines IL-6 and TNF-α in experimental NEC. These confirm that IRF5 is critical in establishing inflammatory phenotypes in NEC.

Our study shows that *Irf5* deficiency in myeloid cells significantly, but not fully, blocks NEC development, indicating that mechanisms other than IRF5 activation may be involved in the pathogenesis of NEC. The microbial ecology of neonates who develop NEC, which involves numerous gram-negative pathogens with high levels of wall LPS, differs from that of control infants.^46,47^ Whether IRF5 expression in myeloid cells alters the gut microbiome to regulate the pathogenesis of NEC needs to be explored in the future.

One of the limitations of the study is the use of intestinal tissues from full-term newborns with congenital intestinal atresia as the control group. Infants with intestinal atresia usually undergo surgery within a few days after birth, when the small intestinal tissue at the edge of the atresia segment is relatively normal. However, the control neonates used in this study were not age-matched controls for the neonates with NEC. A model of hypoxia and hypothermia-induced experimental NEC was used in the present study. Although the final outcome of this model is intestinal necrosis, the more proximal events that incite the pathophysiologic cascade (hypoxia and cold stress) have not been clearly shown to recapitulate the events seen in humans.^43^

To sum up the findings, the present study investigated the effect of myeloid-specific *Irf5* ablation on NEC. Our data strongly suggest that IRF5 promotes M1 macrophage polarization, which leads to overactive inflammation, IECs apoptosis and barrier dysfunction, and exacerbates NEC. Inhibiting IRF5 in macrophages is a potential therapeutic target in NEC.

## Supplementary information

Supplementary information
