# Supplementary material for: Irf5 deficiency in myeloid cells prevents necrotizing enterocolitis by inhibiting M1 macrophage polarization
Source: Mucosal Immunol. 2019 May 13;12(4):888–96. doi: 10.1038/s41385-019-0169-x (PMC7746522; doi:10.1038/s41385-019-0169-x)
Supplement: Supplementary file 1 — Supplementary information [file 41385_2019_169_MOESM1_ESM.docx]

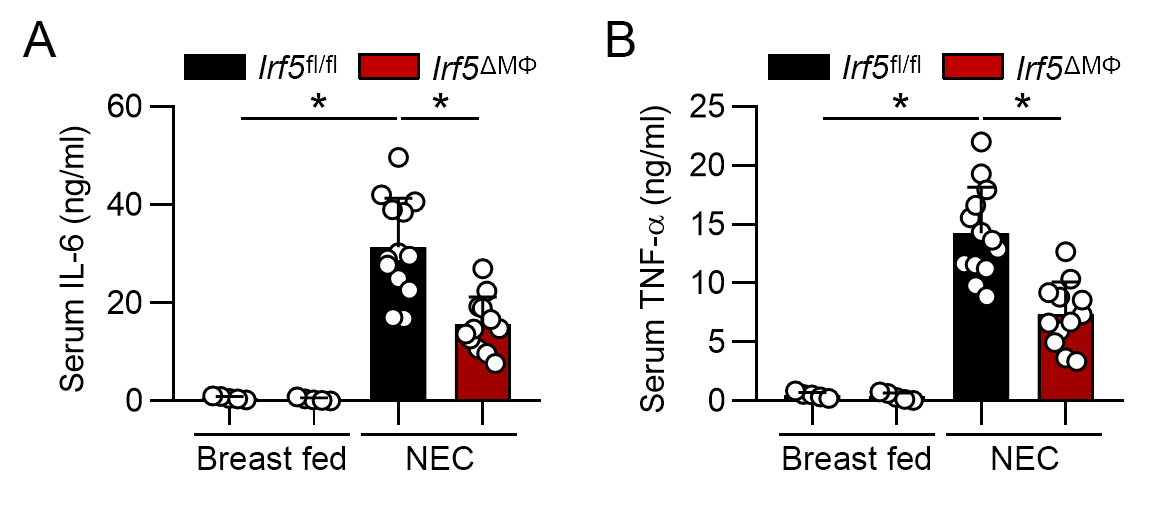


**Supplemental Figure 1. *Irf5* deletion in myeloid cells suppresses systematic inflammation in experimental NEC.** ELISA analysis showed that NEC challenging significantly increased serum IL-6 **(A)** and TNF-α levels **(B)** compared to the breast-fed controls. *Irf5* deletion in myeloid cells significantly suppressed the effects. (n=5 in *Irf5*^fl/fl^ + breast fed; n=5 in *Irf5*^ΔMΦ^ + breast fed; n=13 in *Irf5*^fl/fl^ + NEC; n=12 in *Irf5*^ΔMΦ^ + NEC. *P<0.05)


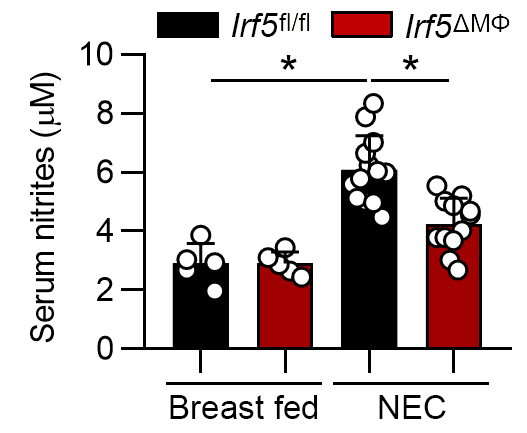


**Supplemental Figure 2. Myeloid-specific ablation of *Irf5* reduces serum iNOS products.** NEC challenging significantly increased serum nitrites compared to the breast-fed controls. *Irf5* deletion in myeloid cells significantly suppressed the effect. (n=5 in *Irf5*^fl/fl^ + breast fed; n=5 in *Irf5*^ΔMΦ^ + breast fed; n=13 in *Irf5*^fl/fl^ + NEC; n=12 in *Irf5*^ΔMΦ^ + NEC. *P<0.05)


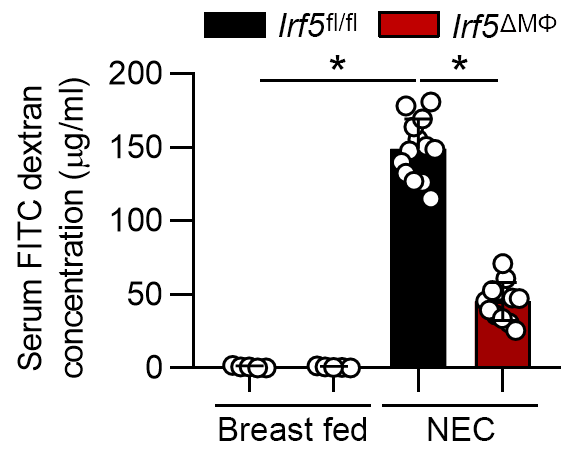


**Supplemental Figure 3. Myeloid-specific deficiency of *Irf5* prevents intestinal barrier dysfunction in NEC.** NEC challenging significantly increased serum FITC dextran leakage compared to the breast-fed controls. IRF5 deletion in myeloid cells significantly suppressed the effect. (n=5 in *Irf5*^fl/fl^ + breast fed; n=5 in *Irf5*^ΔMΦ^ + breast fed; n=13 in *Irf5*^fl/fl^ + NEC; n=12 in *Irf5*^ΔMΦ^ + NEC. *P<0.05)

**Supplemental Table 1. Clinical characteristic in patients with NEC versus control**

|  | No. | Sex | Gestational  age | Birth weight  (g) | Age of onset of NEC (d) | Age at  surgery (d) | Clinical signs | Radiographic findings | Bell stage | Pathology results | Indication for surgery |
| --- | --- | --- | --- | --- | --- | --- | --- | --- | --- | --- | --- |
| Control | 1 | F | 40w+5d | 3290 | / | 1 | Emesis | Ileus | N/A | Complete jejunal atresia |  |
|  | 2 | M | 38w | 3250 | / | 3 | Abdominal distention, emesis | Ileus | N/A | Complete small intestinal atresia |  |
|  | 3 | F | 39w | 2470 | / | 3 | Abdominal distention, emesis | Ileus | N/A | Complete small intestinal atresia |  |
|  | 4 | M | 38w | 3600 | / | 3 | Abdominal distention, emesis | Ileus | N/A | Complete small intestinal atresia |  |
|  | 5 | M | 37w+4d | 3050 | / | 2 | Abdominal distention | Ileus | N/A | Complete small intestinal atresia |  |
| NEC | 1 | F | 29w+3d | 1400 | 11 | 17 | Abdominal distention | Pneumatosis intestinalis | III | Hemorrhagic necrotizing enteritis | Pneumatosis intestinalis |
|  | 2 | M | 31w | 1510 | 8 | 13 | Abdominal distention | Intestinal wall edema, intestinal distention | III | Hemorrhagic necrotizing enteritis | Fixed loop of bowel |
|  | 3 | M | 29w+5d | 1500 | 14 | 37 | Abdominal distention | Ileus | III | Hemorrhagic necrotizing enteritis with granulation tissue hyperplasia | Fixed loop of bowel |
|  | 4 | M | 27w+4d | 1265 | 10 | 11 | Abdominal distention | Intestinal wall edema,  intestinal distention | III | Hemorrhagic necrotizing enteritis | Fixed loop of bowel |
|  | 5 | M | 26w | 970 | 72 | 80 | Abdominal distention | Pneumoperitoneum | III | Hemorrhagic necrotizing enteritis | Pneumoperitoneum |
|  | 6 | M | 31w+2d | 1660 | 22 | 23 | Abdominal distention | Intestinal wall edema | III | Hemorrhagic necrotizing enteritis | Fixed loop of bowel |
|  | 7 | M | 27w+4d | 1200 | 42 | 68 | Abdominal distention | Pneumoperitoneum | III | Hemorrhagic necrotizing enteritis with necrotic rupture | Pneumoperitoneum |
|  | 8 | F | 26w+6 | 940 | 27 | 29 | Abdominal distention | Pneumoperitoneum | III | Hemorrhagic necrotizing enteritis | Pneumoperitoneum |
|  | 9 | F | 32W | 1750 | 8 | 44 | Abdominal distention | Ileus | III | Hemorrhagic necrotizing enteritis | Fixed loop of bowel |
|  | 10 | F | 31W+1d | 1620 | 8 | 10 | Abdominal distention | Pneumoperitoneum | III | Hemorrhagic necrotizing enteritis | Pneumoperitoneum |

**Supplemental Table 2. Primers for quantitative PCR.**

| Gene name | Sequence (5' to 3') |
| --- | --- |
| *Nos2-F* | CAAGCACCTTGGAAGAGGAG |
| *Nos2-R* | AAGGCCAAACACAGCATACC |
| *Tnf-F* | CATGAGCACAGAAAGCATGATCCG |
| *Tnf-R* | AGCAGGAATGAGAAGAGGCTGAG |
| *Il1b-F* | CCCAACTGGTACATCAGCAC |
| *Il1b-R* | TCTGCTCATTCACGAAAAGG |
| *Il12a-F* | GTCTTAGCCAGTCCCGAAAC |
| *Il12a-R* | GGTCCCGTGTGATGTCTTC |
| *Il6-F* | ACAACCACGGCCTTCCCTACTT |
| *Il6-R* | CACGATTTCCCAGAGAACATGTG |
| *Il4ra-F* | TCTGCATCCCGTTGTTTTGC |
| *Il4ra-R* | GCACCTGTGCATCCTGAATG |
| *Mrc1-F* | CTCTGTTCAGCTATTGGACGC |
| *Mrc1-R* | CGGAATTTCTGGGATTCAGCTTC |
| *Retnla-F* | CCAATCCAGCTAACTATCCCTCC |
| *Retnla-R* | ACCCAGTAGCAGTCATCCCA |
| *Arg1-F* | CTCCAAGCCAAAGTCCTTAGAG |
| *Arg1-R* | AGGAGCTGTCATTAGGGACATC |
| *Gapdh-F* | AGGTCGGTGTGAACGGATTTG |
| *Gapdh-R* | TGTAGACCATGTAGTTGAGGTCA |

**Supplemental Table 3. Primers for ChIP quantitative PCR.**

| Gene name | Sequence (5' to 3') |
| --- | --- |
| *Ccl4-F* | CCCCCTCCAACATGCACCCCACT |
| *Ccl4-R* | TGCAGGAAGGAAAACGGAATCTCATG |
| *Ccl5-F* | TTGCCTTAAGACAACAGCTCCCTGCT |
| *Ccl5-R* | CCCCCCCAGCCCCAGGACTT |
| *Tnf-F* | TGGGGACGACGGGGAGGAGATT |
| *Tnf-R* | TGGGGAAGAGGGCGGGGAA |
| *Il12b-F* | AGCCATTGCCGCCTCTATTCACCTTA |
| *Il12b-R* | GAAGTTCCTCCCTCCCCCCCTTG |
